# Effects of Alcohol Consumption on Oxidative Stress in a Sample of Patients Recruited in a Dietary Center in a Southern University Hospital: A Retrospective Study

**DOI:** 10.3390/medicina58111670

**Published:** 2022-11-18

**Authors:** Daniela Metro, Francesco Corallo, Francesco Fedele, Martina Buda, Luigi Manasseri, Viviana Lo Buono, Angelo Quartarone, Lilla Bonanno

**Affiliations:** 1 Department of Biomedical and Dental Sciences and Morphofunctional Imaging, University of Messina, 98125 Messina, Italy; 2 IRCCS Centro Neurolesi “Bonino-Pulejo”, 98124 Messina, Italy; 3 Department of Human Pathology in Adulthood and Childhood “G. Barresi”, University of Messina, 98125 Messina, Italy

**Keywords:** alcohol consumption, dietary pattern, glutathione, lipid peroxidation, malondialdehyde, oxidative stress

## Abstract

*Background and objectives*: The aim of this retrospective study was to evaluate the effects of alcohol consumption on oxidative stress. *Materials and Methods*: The study was conducted by analyzing the increase in lipid peroxidation, the reduction of antioxidant defenses and the alteration of the oxidation/antioxidant balance after the administration of ethanol in 25% aqueous solution (*v*/*v*) at a concentration of 0.76 g/kg of body weight daily in two doses for 3 days. The changes in oxidative stress indices were investigated by standard methods previously described. *Results*: Ethanol administration has determined a significant increase in plasma levels of lipid hydroperoxide (LOOH), malonilaldehyde (MDA) and oxidized glutathione (GSSH), and a decrease in total antioxidant capacity (TAC), reduced glutathione (GSH) and GSH/GSSH ratio. *Conclusions*: In the proposed experimental condition, the excessive and repeated consumption of ethanol causes oxidative damage, as shown by the increase in lipid peroxidation, the reduction of antioxidant defenses and the alteration of the oxidation/antioxidant balance, which, at least in part, are responsible for the harmful effects of excess ethanol.

## 1. Introduction

Ethanol, contained in alcoholic beverages, is a substance of nutritional interest but no nutrient value, that, while providing energy (7 kcal/g), has no specific functional and/or metabolic purposes, as described by The Food and Nutrition glossary [1]. Instead, it is a potentially toxic molecule for organisms and represent a high social risk factor, because its abuse can cause important organic and psychological damage; high ethanol intake is associated with an increased risk of cardio-cerebrovascular diseases, liver disease, gastrointestinal diseases and some forms of cancer; alcohol induces oxidative stress either by direct action by increasing the production of oxidizing chemical species or by indirect action by reducing the antioxidant capacity of the cell [2].

The studies on the relationship between alcoholic beverages and health highlight a typical J or U curve, that describes the relationship between ethanol intake and mortality [3], cardiovascular diseases [4,5] and diabetes [6].

A moderate alcohol consumption has been associated with a healthy protective effect and to lower risk of important diseases such as ischemic heart disease, ischemic stroke, osteoporosis and diabetes [7,8]. This trend has been confirmed by some meta-analyses [9,10,11] that showed how low consumption of alcoholic beverages (up to 10 g/day for women and 20 g/day for men) is associated with a lower incidence of vascular events and mortality.

However, even if a low consumption of ethanol is associated with health benefits, alcohol could represent, even in a low amount, a risk of cancer [12,13].

A high consumption of alcohol (>30 g/day), on the other hand, increases the risk of various diseases, such as hepatopathies (alcoholic liver disease and cirrhosis), digestive problems (gastritis, esophagus and stomach ulcers and pancreatitis), hypertension and cardiovascular diseases [14], complications of diabetes mellitus [15] and cancer; alcoholism increases the risk of mouth, esophagus, liver, colon and breast cancer [16], fetal alcohol syndrome [17] and degenerative neuropathies [18].

In summary, the abuse of ethyl alcohol causes over 200 diseases and is the third highest risk factor for premature death and disability and the first highest risk factor in the age group under 25 [19,20].

There is still no awareness of the damage, while society tolerates or even encourages its consumption, risking excessive use or a real addiction in young people, as the excessive consumption of alcohol acts on various brain circuits, altering their functioning [21].

The state of intoxication also impairs the ability to make decisions, increasing the risk of exposing oneself to dangerous situations, such as unprotected sexual intercourse, incurring traffic accidents, being the victim of violence or exhibiting aggressive behavior [22,23,24].

The causes of alcohol damage are varied, from genetic and biological factors to psychosocial elements, from erroneous educational models to stress and trauma. Among the biological factors we must remember oxidative stress, the subject of our work.

The health authorities (WHO-2004 Recommendation, WCRF/AICR 2007 USDA 2010 guidelines) consider it appropriate to provide information on daily thresholds not to be exceeded (the only aspect for which we have guidelines), but there is no recommendation: only people over 18 years old can drink, and the maximum threshold must not exceed one alcoholic unit per day for women and two units for men, to be consumed with a meal.

A correlation between alcohol abuse and the production of Reactive Oxygen Species (ROS), reduction of antioxidant defenses, alteration of the balance between oxidants and antioxidants and oxidative damages to proteins, lipids, carbohydrates and cell nucleic acids has been shown. In contrast, experience have shown that green tea [25], caffeine [26] and Aloe Vera [27] are able to reduce oxidative stress. On the other hand, the Mediterranean diet, characterized by vegetables, fruit, cereals, nuts, olive oil and wine consumption is particularly rich in antioxidants [28,29,30,31,32] and molecules of nutritional interest [33,34,35,36]. However, recent studies have shown that, in various populations, the adherence to the traditional Mediterranean diet is decreasing [37,38]. Ethanol consumption promotes oxidative stress in cells by inducing morphological and functional alterations in target organs such as the brain. Ethanol, administered to mammals, induces neurotoxic effects in specific areas of the central nervous system such as the limbic system, cortex and striatum. These effects occur mainly at the level of the cholinergic system, where neurodegeneration can be observed following the treatment of both young and adult or elderly animals [38]. The literature is very heterogeneous on the identification of blood markers to see the consequences in this population type. It is also complicated to be precise about the consequences of biomarkers of oxidative stress, because only in a limited percentage of diseases have a range of different biomarkers been used, and different biomarkers have been used to study different diseases. In addition, biomarkers are often measured by nonspecific methods, while specific methodologies are often too sophisticated or laborious for routine clinical use. In our study, we decided to consider some of them, relying mainly on what the literature has suggested [39].

As we have seen, the literature has focused more on the misuse of alcohol from the perspective of preventative healthcare; the aim of our study was to investigate the effects of ethanol on oxidative stress in healthy volunteers that following a balanced eating plan according to individual needs as indicated by the Italian Society of Human Nutrition.

Finally, several studies have highlighted that there is not quantity of alcoholic beverage that can be consumed, even a moderate one, which is not a risk. In fact, it has been shown that moderate intake of ethanol exposes you to the risk of cancer and chronic degenerative diseases, which seriously affect quality of life due to factors such as diabetes and metabolic, cardiovascular and neurodegenerative diseases that have inflammation in common. The latter is considered potentially capable of triggering and promoting the development of the aforementioned chronic degenerative diseases. Oxidative stress, which is the subject of our study, has been proposed to be among the “trigger” factors.

## 2. Materials and Methods

This was a retrospective study conducted on a sample of 31 outpatients attending the Autonomous Service of General Dietetics of the University Hospital of Messina from January 2021 for 1 year.

Inclusion criteria were: a weight at least 77 kg, BMI at least 24, non-smokers, teetotalers and with specific dietary habits (following a balanced eating plan according to individual needs as indicated by the Italian Society of Human Nutrition, LARN—Levels of Assumption Reference of Nutrients and Energy for the Italian population, IV revision, 2014).

Ethanol was administered orally in a 25% aqueous solution (*v*/*v*) at a concentration of 0.76 g/kg of body weight per day in two doses (0.38 g/kg at lunch and 0.38 g/kg at dinner) for 3 days. Indicators of oxidative stress were analyzed in plasma before and after ethanol intake, evaluating both oxidative damage and antioxidant defenses.

The oxidative damage, due to radical species, was assessed through the quantification of lipid peroxidation and the determination of LOOH (μmol/L) and MDA (nmol/mL) plasma levels [23].

The variation of antioxidant defenses was evaluated by the analysis of TAC (μmol/L), GSH (μmol/L) and GSSH (μmol/L) levels.

Lipid peroxidation was quantified by assessing the oxidative state of the plasma through determination of the levels of lipid hydroperoxides (LOOH, µmol/L) by means of spectrophotometric technique analysis, and malondialdehyde (MDA) levels by high-performance liquid chromatography (HPLC). For LOOH, we used the Oxis Bioxytech^®^ LPO-560™ Assay (Oxis International, Inc., Portland, OR, USA). This assay is based on the oxidation of ferrous ions (Fe^2+^) to ferric ions (Fe^3+^) by hydroperoxides under acidic conditions. Ferric ions then bind with the indicator dye, xylenol orange, and form a colored complex. The absorbance of the complex was measured at 560 nm. For MDA measurement, 250 μL serum was added to 50 μL NaOH 6 M and then incubated at 60 °C in a water bath for 30 min. Afterwards, proteins were precipitated with 125 μL 35% perchloric acid (*v*/*v*), with subsequent centrifugation at 2800 rpm for 10 min. Next, 250 μL of the supernatant was transferred into an Eppendorf tube and mixed with 25 μL DNPH, which had been prepared as a 5 mM solution in 2 M hydrochloric acid. This mixture was incubated for 30 min at room temperature in the dark and 50 μL was analyzed by HPLC.

The total antioxidant power (TAC, μmol/L) was determined by a colorimetric technique, using a commercial kit, DIACRON (Grosseto, Italy).

The modulation of antioxidant defenses was determined by analyzing plasma levels of reduced glutathione (GSH, μmol/mL), oxidized glutathione (GSSH, μmol/mL) and GSH/GSSH ratio. GSH and GSSH were measured by means of HPLC. This extraction procedure requires that blood samples are collected in vacutainer tubes containing K3-EDTA. After collection, 100 μL fresh blood was mixed with 12 μL phosphate buffer 10 mmol/l, pH 7.2 (for free GSH), or 12 μL phosphate buffer 10 mmol/L, pH 7.2, containing 10 mM N-ethylmaleimide (for oxidized GSH). One hundred μL of this mixture was hemolyzed by adding 900 μL distilled water and immediately deproteinized by adding 200 μL sulfosalicylic acid (12% volume). The content of GSH was assessed in the acid-soluble fraction [25].

### Statistical Analysis

Continuous variables were expressed as mean ± standard deviation. A nonparametric analysis was carried out because the results of the Shapiro normality test indicated that most of the target variables were not normally distributed. The numerical data are presented in median, and first-third quartile in non-normal distribution. To observe time-varying differences in the whole sample, the Wilcoxon signed-rank test was applied. Subsequently, the sample was divided into two groups by gender. The Mann–Whitney U test and Wilcoxon signed-rank test were used for inter and intra-group analysis, respectively. Analyses were performed using an open source R3.0 software package. A 95% confidence level was set with a 5% alpha error. Statistical significance was set at *p* < 0.05.

## 3. Results

Demographic and clinical characteristics of the sample are reported in Table 1. The Wilcoxon signed-rank test shows highlighted significant differences in clinical variables (*p* < 0.01) between T0 and T1 (Table 1).

In Table 2 reports the clinical variables of the sample divided into two groups by gender. Intra-group analysis showed significant differences: in particular, in male groups, there was a significative difference in GSH (*p* = 0.004) and in GSSH (*p* = 0.05), while in the female groups, a significant difference in MDA (*p* < 0.001), TAC (*p* < 0.001) and GSH (*p* < 0.001) were found (Table 2).

Inter-group analysis highlighted significant differences between groups with respect to each variable (Table 2).

From the data obtained, it is evident that the administration of ethanol has determined significant increase in plasma levels of lipid hydroperoxide (LOOH), significant increase in plasma malonilaldehyde (MDA) levels, decrease of total antioxidant capacity (TAC), reduction of reduced glutathione (GSH) and increase of oxidized glutathione (GSSH).

## 4. Discussion

The association between alcohol consumption, oxidative stress and pathologies is reported in our work and other studies [40]. It is well documented that LOOH and MDA are mediators and inducers of oxidative stress. Plasma TAC is used as a biomarker for non-enzymatic antioxidant status and oxidative stress [40].

GSH, the most abundant non-protein thiol that protects against oxidative stress [41], is considered a biomarker of redox imbalance at the cellular level [42]. It represents one of the main endogenous mechanisms of protection of the lipoperoxidative phenomenon. Numerous studies have shown that both acute and chronic ethanol intake are followed by an evident decrease in GSH levels. This depletion could be related to the detoxifying function of GSH in relation to the harmful ethanol action. GSH limits the production of lipoperoxides through the oxidation of GSH to GSSH in the presence of GSH peroxidase [43] and the inactivation the free radicals generated during ethanol metabolism.

Furthermore, an additional cause of GSH decrease has been observed in alcohol intoxication phenomena; in fact, ethanol directly inhibits the synthesis of GSH [44].

In the pathogenesis of alcohol damage, the central role is played by lipid peroxidation, which causes an increased production of ROS, free radicals and non-radicals.

In fact, several pieces of evidence show that the excessive consumption of ethanol induces the production of different ROSs, which are responsible for alcoholic liver disease (ALD). Excessive consumption of ethanol can cause mitochondrial damage, induction of CYP2E1, ethanol-induced activation of Kupffer cells and depletion of mitochondrial and cytosolic glutathione. These routes lead to the production of a variety of ROSs in the liver, such as superoxide, H_2_O_2_ and hydroxyl radical [45,46]. The susceptibility of tissues to alcohol-induced damage is related to their function and the way by which they are exposed to alcohol. Chronic alcohol use causes the induction of P450 2E1. This enzyme is one of the components that participates in microsomal oxidative mechanisms capable of generating ROS.

Briefly, ROS can cause: cholesterol, PUFA and protein oxidation in lipoproteins molecules; the oxidation of -SH groups of enzymes, the formation of covalent bonds with other molecules and proteolysis in proteins; the oxidative degradation of connective tissue in proteoglycans; mutations in nucleic acids; and in carbohydrates, oxidation and polymerization [28].

Several studies highlight the involvement of an excessive ROS amount as a trigger for different pathologies. ROS and oxidative stress are involved in aging [46,47] and in various diseases: diabetes mellitus [48,49], atherosclerosis [50], rheumatoid arthritis [51], Alzheimer’s disease [52], Parkinson’s disease [53], cancer [16] and some liver diseases. As a result, excessive consumption of ethanol can be associated with an increase in the incidence of these diseases.

The oxidative stress observed in alcohol-related hepatopathies has been highlighted by numerous studies. It has been shown that oxidative stress induces inflammation, hepatic steatosis, fibrosis and apoptosis [54,55].

Inflammation is one of the most frequent outcomes of oxidative stress. It has been suggested that oxidative stress may stimulate the expression of chemokines and cytokines, [56] and excessive inflammation may cause cellular damage.

Chronic ethanol consumption is associated with hypertension and atherosclerosis. In fact, it has been pointed out that the excessive consumption of ethanol increases the levels of angiotensin II, oxidative vascular stress and arterial pressure, with a dependent mechanism of type I angiotensin receptors (AT1) [57]. In addition, it has been shown that the sympathetic nervous system is also a mediator of the cardiovascular effects of ethanol. One study found that in rats, nebivolol, a selective inhibitor of B1-adrenergic receptors, prevents vascular oxidative stress and ethanol-induced hypertension [58].

Moreover, free radicals play an important role in the pathogenesis of atherosclerosis. It is widely demonstrated that the peroxidation of lipids and apolipoproteins, induced by free radicals, is the main cause of conformational modifications of both low-density lipoproteins (LDL) and high-density lipoproteins (HDL), and that these oxidized lipoproteins (oxLD and oxHDL) are one of the main causes contributing to the formation of arteriosclerotic lesions [34].

The results of this study were very encouraging, because they are very indicative of changes in plasma levels even with limited alcohol consumption. This could encourage future research even on the level of primary prevention, which takes on very important meanings today. The limitations of the study, however, imply that the variables to be investigated could be increased, and also the group under study could be subjected to more follow-up, and the sample number could be increased. From the data obtained, it is important to emphasize the importance of ethanol on plasma levels and lipid hydroperoxide (LOOH), a significant increase in plasma levels of malonylaldehyde (MDA), a decrease in total antioxidant capacity (TAC), a decrease in reduced glutathione (GSH) and an increase in oxidized glutathione (GSSH).

Furthermore, it is widely shown that oxidative stress is correlated with multiple components of metabolic syndrome: diabetes mellitus, hypertension, obesity, dyslipidemia and inflammation. Evaluation of oxidative stress in individuals with metabolic syndrome may contribute to the identification of patients with an increased risk of metabolic and cardiovascular complications [59]. Lipid peroxidation in diabetes induces secondary chronic complications, including atherosclerosis and nervous disorders [59,60].

Increased levels of MDA and reduced levels of GSH in plasma and in many tissues were reported in diabetic patients [61,62].

## 5. Conclusions

In the proposed experimental condition, the excessive and repeated consumption of ethanol causes oxidative damage, as shown by the increase of lipid peroxidation, the reduction of antioxidant defenses and the alteration of the oxidation/antioxidant balance, which, at least in part, are responsible for the harmful effects of excess ethanol, as previously described [38].

## Figures and Tables

**Table 1:** Socio-demographic and clinical characteristics of sample (% frequency).

	T0	T1	*p*
Age	24.6 ± 2.4	-	
Gender			
Male	31 (59.6)	31 (59.6)	
Female	21 (40.4)	21 (40.4)	
Education			
LOOH	2.1 (1.74–2.2)	4.2 (3.9–4.42)	<0.001 *
MDA	0.95 (0.88–1.0)	2.35 (2.3–2.6)	<0.001 *
TAC	407.5 (390–418.5)	230 (225–244)	<0.001 *
GSH	12.1 (10.77–14.9)	6.45 (5.9–6.8)	<0.001 *
GSSH	2.81 (2.55–3.09)	4.3 (3.9–4.7)	<0.001 *

* *p* < 0.05

**Table 2:** Intra and Inter analysis.

		Male	Female	
		Median (I–III Quartile)	Median (I–III Quartile)	*p*
LOOH	T0	2.15 (1.74–2.20)	1.98 (1.6–2.1)	<0.001 *
	T1	4.1 (3.9–4.4)	4.3 (4.1–4.6)	<0.001 *
	*p*	0.17	0.1	
MDA	T0	0.95 (0.87–1.01)	0.95 (0.9–0.99)	<0.001 *
	T1	2.3 (2.26–2.35)	2.7 (2.55–2.8)	<0.001 *
	*p*	0.84	<0.001 *	
TAC	T0	412 (390–418)	407 (395–420)	<0.001 *
	T1	238 (228–249)	225 (219–230)	<0.001 *
	*p*	0.65	0.0002 *	
GSH	T0	13.9 (11.05–15.9)	11.4 (10.3–12.1)	<0.001 *
	T1	6.7 (6.35–6.85)	5.9 (5.8–6.4)	<0.001 *
	*p*	0.004 *	0.0005 *	
	T0	2.85 (2.7–3.1)	2.64 (2.45–2.97)	<0.001 *
GSSH	T1	4.5 (4.12–4.65)	4.1 (3.7–4.7)	<0.001 *
	*p*	0.05 *	0.2	

* *p* < 0.05

## Data Availability

https://zenodo.org/record/7323224#.Y3N_Q-TMKUk.
